# Nanofabrication of Bulk Diffraction Nanogratings via Direct Ultrashort-Pulse Laser Micro-Inscription in Elastomers and Heat-Shrinkable Polymers

**DOI:** 10.3390/nano13081347

**Published:** 2023-04-12

**Authors:** Vladimir Kesaev, Alexey Rupasov, Nikita Smirnov, Petr Pakholchuk, Sergey Kudryashov, Galina Odintsova

**Affiliations:** 1 Lebedev Physical Institute, Moscow 119991, Russia; 2 Engineering Physics Faculty, ITMO University, St. Petersburg 197101, Russia

**Keywords:** thermally shrinkable polymers (thermoplastics), elastomers, ultrashort-pulse laser, direct laser inscription, bulk diffraction gratings

## Abstract

Optical-range bulk diffraction nanogratings were fabricated via challenging direct inscription by ultrashort (femtosecond, fs) laser pulses inside heat-shrinkable polymers (thermoplastics) and VHB 4905 elastomer. The inscribed bulk material modifications do not emerge on the polymer surface, being visualized inside the materials by 3D-scanning confocal photoluminescence/Raman microspectroscopy and by the multi-micron penetrating 30-keV electron beam in scanning electron microscopy. The laser-inscribed bulk gratings have multi-micron periods in the pre-stretched material after the second laser inscription step, with their periods continuously reduced down to 350 nm on the third fabrication step, using thermal shrinkage for thermoplastics and elastic properties for elastomers. This three-step process allows facile laser micro-inscription of diffraction patterns and their following controlled scaling down as a whole pattern to pre-determined dimensions. In elastomers, utilizing the initial stress anisotropy, the post-radiation elastic shrinkage along the given axes could be precisely controlled until the 28-nJ threshold fs-laser pulse energy, where elastomer deformation ability is dramatically reduced, producing wrinkled patterns. In thermoplastics, the fs-laser inscription does not affect their heat-shrinkage deformation up to the carbonization threshold. The measured diffraction efficiency of the inscribed gratings increases during the elastic shrinkage for the elastomers and slightly decreases for the thermoplastics. High 10% diffraction efficiency was demonstrated for the VHB 4905 elastomer at the 350 nm grating period. No significant molecular-level structural modifications were observed by Raman micro-spectroscopy in the inscribed bulk gratings in the polymers. This novel few-step method paves the way for facile and robust ultrashort-pulse laser inscription of bulk functional optical elements in polymeric materials for diffraction, holographic and virtual reality devices.

## 1. Introduction

The ongoing progress in nanofabrication technologies breaks the receding barriers in the integration of functional elements in nanoelectronics and nanophotonics, boosting the modality and functionality of the integrated devices [1,2]. Specifically, the fabrication of relief-based diffraction optical elements (DOE) by traditional holographic and lithographic methods is crucially dependent on the photosensitivity of optical materials in use. For this reason, very often, multiple steps—spin-coating of high-resolution photoresists, their photo-exposure, development and tanning—are required to achieve necessary spatial DOE parameters. In some cases, high-efficiency transparent DOE could be fabricated by imprinting in transparent polymers [1,2], enabling their high diffraction efficiency while requiring special soft materials [3,4,5]. Moreover, during the last decade, the versatility of 2D and 3D direct ultrashort (femtosecond or picosecond, fs or ps) pulse laser inscription on surfaces and in the bulk of transparent materials was demonstrated [6,7,8,9,10,11,12,13,14,15,16,17,18,19,20,21].

Direct ultrashort pulse laser inscription emerges as a facile tool for fabricating optical elements in different materials (metals, semiconductors, dielectrics and polymers [6,7,8,9,10,11]). Fs-laser induced high nonlinear absorption in any possible material provides the necessary energy deposition for the material modification and its high, near-wavelength spatial resolution [10]. Although the spatial resolution of such direct ultrashort pulse laser inscription is commonly limited by the diffraction limit~λ/2, deeply sub-wavelength elements down to tens or hundreds of nanometers could be realized for threshold-like two-photon polymerization [20], high-NA (numerical aperture NA > 1) laser ablation [7,8,9], plasmonic self-organization of sub-wavelength nanogratings in bulk solid dielectrics [10,18]. Meanwhile, auxiliary post-radiation treatment—e.g., etching [17], annealing, etc.—is often required to envision the sub-wavelength details of such laser-inscribed patterns.

Recently, ultrashort laser pulses were utilized for refractive index modification in polymers—both on surfaces and in bulk, producing diffraction gratings [11,12,13,14,15,16]. In these applications, polymeric elastomers and hydrogels appear as perspective materials, supporting their considerable reversible deformations [20,21,22,23]. As a result, different nanophotonic devices—deformable metasurfaces and phase diffraction gratings were demonstrated for such elastoplastic materials [19,20,21,22,23,24,25]. Meanwhile, the relevant mechanical modalities of elastomers—e.g., their many-fold elastic deformation (low bulk modulus ~10^2^–10^3^ kPa), non-linear deformation versus stress and minimal plasticity—were not ultimately harnessed in nanophotonics up to the date.

**Figure 1:**
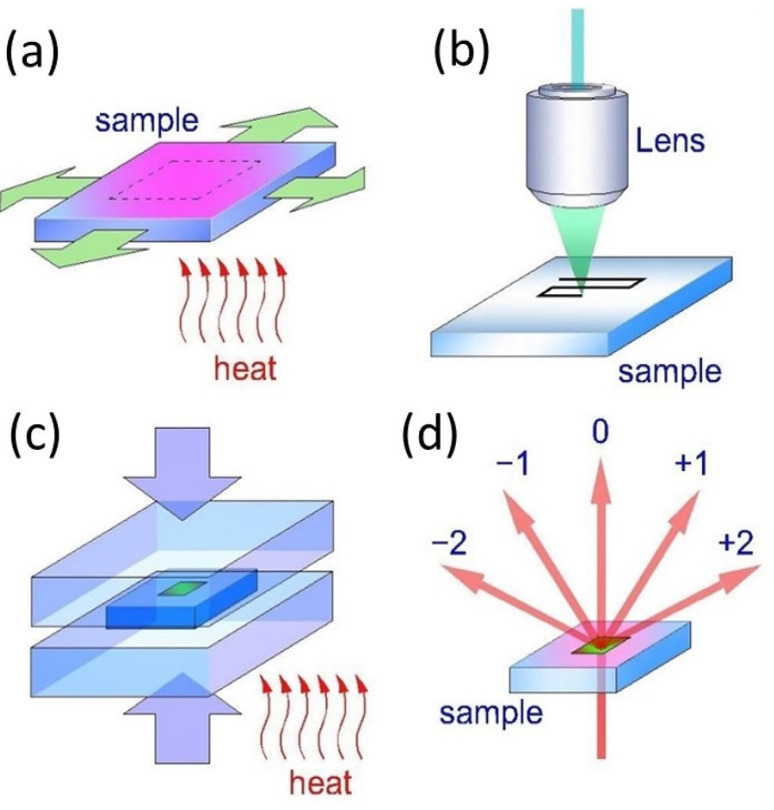
Three-step fabrication of sub-wavelength diffraction gratings in heat-shrinkable polymers: (**a**) pre-stretching of the thermoplastic sheet; (**b**) laser inscription of diffraction micro-grating; (**c**) controllable heat-shrinking down to nanoscale dimensions; (**d**) diffraction test of the downscaled grating.

Similarly, heat-shrinkable (thermoplastic) polymers could potentially be a perspective material platform in nanophotonics [26,27]. Such two-phase materials contain one hyper-elastic but “temperature-independent” phase, working as a “skeleton”, while the other phase appears as a rigid solid amorphous filler at lower temperatures and a strongly deformable liquid at higher temperatures. Such thermoplastic plate could be stretched in two directions at high temperatures (Figure 1a) and then fixed at lower temperatures for a long time in the flat deformed state without external stresses as a pre-requisite sample for laser inscription. Upon laser inscription, the inscribed surface or bulk structures could be strongly downsized via free heat shrinking of the pre-stretched sample by the internal elastic stresses in the absence of external stresses or external deformations. Such heat-shrinkable polymers are commercially available, optically transparent and variable in refractive indexes, anisotropically stretchable and mechanically handling [27,28], to be complementary polymeric materials for direct ultrashort pulse laser inscription in nanophotonics.

In this study, we demonstrated very challenging bulk *nanopatterning* inside polymers via ultrashort pulse laser *micro-scale inscription*, exploring a few pre-stretched elastomers and thermoplastics for direct fs-laser inscription of sub-wavelength one-dimensional bulk diffraction gratings, utilizing their abovementioned shrinking modalities via the three-step fabrication process. Diffraction efficiencies of the resulting (down)scaled gratings were measured, while their buried arrangement was identified by 3D-scanning confocal photoluminescence/Raman micro-spectroscopy and highly penetrating 30-keV electron beam in scanning electron microscopy at the absence of observable AFM-acquired surface relief. Their molecular-level structural states were characterized by 3D-scanning confocal photoluminescence/Raman micro-spectroscopy too.

## 2. Materials and Methods

In our studies, we used commercially available 1 mm thick sheets of thermoplastic polyethylene terephthalate (PET) and polyurethane (PU), and elastomer VHB 4905 (3M, Berlin, Germany). The samples were processed regarding bulk DOE laser-based fabrication in the following way (Figure 1). First, a thermoplastic PET sample was heated to the thermoelastic state at ~80 °C and then three-fold mechanically stretched in the two orthogonal directions with different magnitudes (Figure 1a). The resulting 2D stretched sample was cooled down to room temperature (23 °C) and subjected to laser inscription of bulk diffraction micro-grating at typical depths of 50–100 μm (Figure 1b, for the details see below). Then, the sample was placed and clamped between two polished glass plates and slowly heated (heating rate ≈ 5 ÷ 10 °C/min), exhibiting its continuous heat-shrinking (Figure 1c). The shrinkage was controlled by fast compression of the sample between the glass plates and the following cooling. Finally, the prepared sample was arranged in the acquisition scheme for diffraction measurements (Figure 1d). The diffraction orders were observed for large, micron-scale grating periods, while for smaller periods (<0.5 μm), the diffracted waves were trapped in the samples as waveguide modes. In the latter case, the recently proposed method of minimal diffraction angle was used [28].

**Figure 2:**
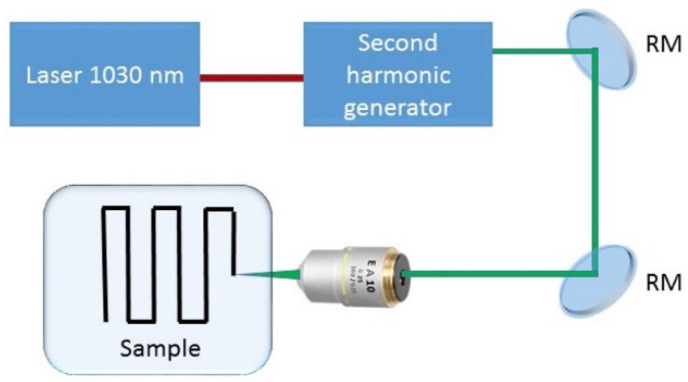
Schematic of the laser inscription scheme, consequently including ultrashort-pulse laser (1030 nm, 0.3 ps), second-harmonic generator (converter to 515 nm radiation), reflective steering mirrors (RM), focusing microscope objective and polymer sample arranged on 3D motorized translation stage for bulk inscription of diffraction gratings.

Laser inscription of the micro-gratings in the polymer sheets was performed by means of a laser micromachining workstation based on a Yb-doped fiber laser Satsuma (Amplitude Systemes, St. Etienne, France). Its second harmonic pulses (central wavelength—515 nm, minimal full-width at a half-maximum ≈ 310–320 fs) were focused at the depths of 50–100 μm under the sample surface (Figure 1b and Figure 2) by 0.65-NA or 0.25-NA objectives into focal spots with their 1/e-intensity radii R_1/e_, equal to 0.7 or 1.9 µm (the evaluated Raleigh lengths ≈ 1.6 or 10 µm), respectively, with the pulse energy in the range of 3.2–14.7 nJ for the thermoplastics and up to 30 nJ for the elastomer. The exposure occurred at the 100 kHz repetition rate with the laser polarization arranged along the grating stripes. Micro-gratings were inscribed inside the stretched thermoplastic samples within the regions of 1.5 × 1.5 mm in size, utilizing the 3D-translation stage (Standa, Vilnius, Lithuania) at the scan speed of 350 µm/s (exposure N_0.25_ = 500 pulses/spot and N_0.65_ = 200 pulses/spot), with their periods of 2, 3 and 10 µm. The elastomer VHB 4905 was four-fold stretched in all directions, while the thermoplastic materials were stretched 2.5 fold along the grating wave vector and up to 1.5 fold in the orthogonal direction.

During the direct laser inscription in the polymer’s refractive properties of the materials in the focal volume is changing, following nanoscale structural transformations just in their molecular packaging [29], thus keeping the material optically transparent. The resulting micro-structure or pattern is composed by separate focal modification pixels, making the required phase pattern with the pulse-energy controlled diffraction efficiency in each pixel. The grating periods and the diffraction efficiency were measured at the 633 nm laser wavelength for the variable shrinkage magnitudes monitored by the grating dimensions (initial size—1.5 × 1.5 mm), varying ×2 and ×4 for the elastomer, ×3.5 and ×4 for the PU sample and ×2 for the PET sample.

**Figure 3:**
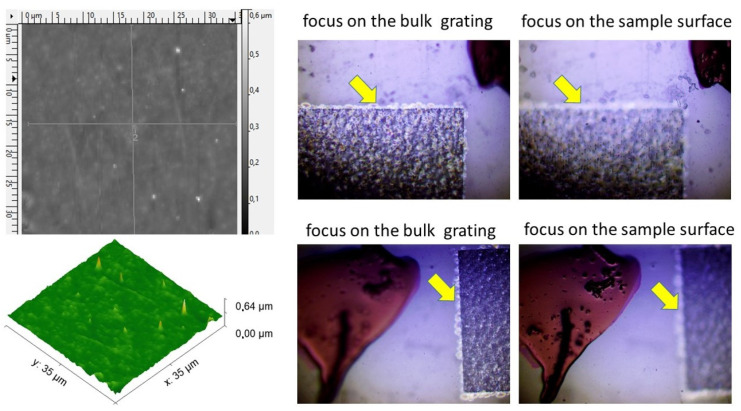
(**left** side) AFM relief maps of the PET sample surface with the micrograting inscribed in its bulk (depth ≈50 μm). (**right**) Optical images of the PET sample with the focus on the micrograting (shown by the yellow arrow) and sample surface.

**Figure 4:**
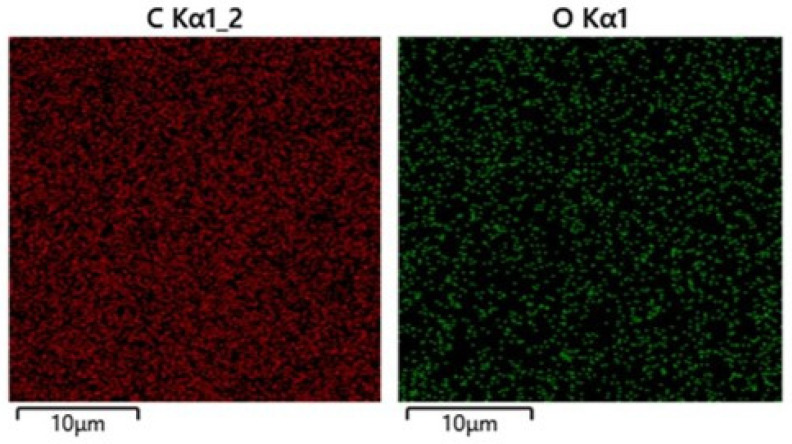
EDX maps of carbon (C) and oxygen (O) on the bulk 3-μm grating inside the PET sample.

The initial and inscribed polymer samples were characterized regarding potential laser-induced surface ablative modification by atomic force microscopy (AFM, Figure 3), using a scanning probe microscope Certus Standard V (Nanoscan, Moscow, Russia) in a contact mode. 3D imaging of bulk buried diffraction gratings, bulk photo- or thermochemical decomposition and, more generally, chemical and structural states of the materials was analyzed by means of a 3D-scanning confocal Raman/photoluminescence microscope-spectrometer Confotec MR 520 (SOL Instruments, Minsk, Belarus) at the 532 nm pump wavelength, using a 0.75-NA objective. Optical images of the micro-gratings were captured by an optical microscope Altami (Altami, St. Petersburg, Russia), while higher-resolution images and chemical characterization of the polymer surfaces regarding possible laser modification effects were obtained by means of a low-vacuum scanning electron microscope VEGA 4 (SEM, TESCAN, Brno, Czechia), equipped by an X-ray energy-dispersion spectroscopy (EDX) module Xplore 15 (Oxford Instruments, Birmingham, UK), as shown in Figure 4 below. Surprisingly, the utilized 30 keV electron beam of the SEM microscope enabled visualization of the buried diffraction gratings, owing to its high penetration length (~10 μm) in the low-density low-mass carbon materials (see for the details ref. [30]).

## 3. Experimental Results and Discussion

**Figure 5:**
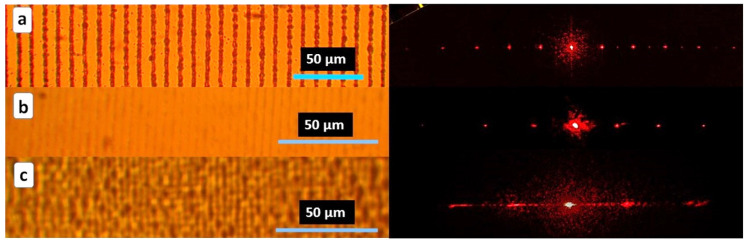
(**left**) Optical images of bulk diffraction microgratings in PU sample (**a**–**c**) and (**right**) their optical diffraction patterns for 633 nm laser light source. Thermal shrinkage by (**a**) ×1 (initial); (**b**) ×3.5 and (**c**) ×4.

One bulk diffraction grating with a period of 10 μm was inscribed by fs-laser pulses inside the thermoplastic PU sample (Figure 5a). Its heat shrinkage is shown in Figure 5b,c, demonstrating not only the strongly reduced spatial periods of 4 and 2.8 μm but also the increased angles between the diffracted 633 nm laser beams of the same order.

Meanwhile, the optical diffraction efficiency of the bulk PU diffraction gratings diminished versus the period (see below); very possibly, the structural relaxation occurred during the shrinkage, diminishing also the additional strong birefringence in the modified region, along with the main artificial stress birefringence.

**Figure 6:**
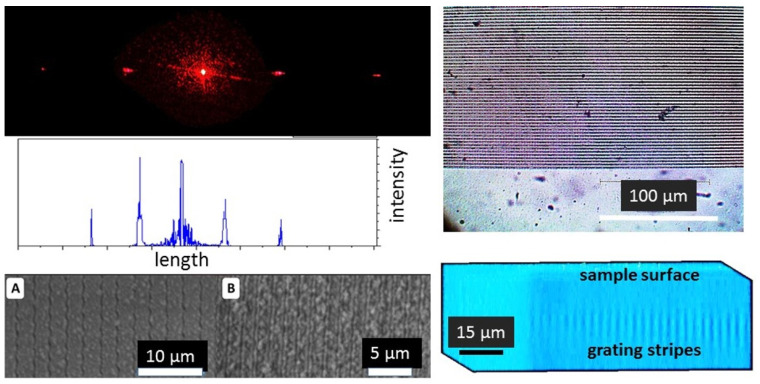
**Right** side: (**top**, **middle**) Optical diffraction pattern and its angular spectrum for 633 nm laser light source produced by the bulk 3 μm diffraction grating in PET sample; (**bottom**) SEM images of bulk diffraction microgratings in PET sample with 3 μm ((**A**), initial) and 1.7 μm ((**B**), shrunk by 2×) periods. **Left** side: (**top**) Optical image of the buried initial diffraction micrograting; (**bottom**) Side-view image of the initial diffraction micrograting under the surface visualized by 3D-scanning confocal Raman/photoluminescence micro-spectroscopy at the 570 nm wavelength.

In the other thermoplastic PET sample, the initial bulk diffraction micro-grating with the period of 3 μm visualized by optical microscopy, SEM and 3D-scanning confocal Raman/photoluminescence micro-spectroscopy was transformed into the other one with the reduced period of 1.7 μm (Figure 6). SEM visualization indicates that the grating stripes are not perfectly straight, while the modified material is non-homogeneous on the sub-micrometer scale. Meanwhile, these circumstances do not affect the acquired optical diffraction patterns, exhibiting no observable noise for these reasons and demonstrating the robustness of the laser-inscription-based fabrication procedure.

**Figure 7:**
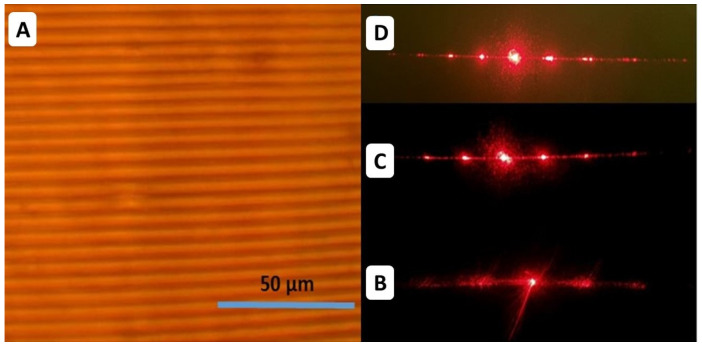
(**left**) Optical image of bulk 10-μm diffraction micrograting in the elastomer VHB 4905 (**A**) and (**right**) the optical diffraction patterns for 633 nm laser light of the initial grating ((**B**), pre-stretched), and of downscaled 5-μm (**C**) and 2.5-μm (**D**) gratings, respectively.

In contrast to the thermoplastic polymers, the elastomer VHB 4905 does not support the heat-shrinkage modality and for this reason it was anisotropically and mechanically pre-stretched in two orthogonal directions. As a result, the laser-inscribed bulk grating period could be then increased or decreased by the following external deformation. In Figure 7 below, the inscribed bulk diffraction grating, possessing the 10 μm period and the sinusoidal profile is shown along with its 633 nm diffraction pattern and the 633 nm diffraction patterns of the mechanically relaxed pre-stretched material. Surprisingly, the two-fold shrinkage of the bulk diffraction grating resulted only in the 7.5-μm period, while the four-fold shrinkage yielded in the 4.5-μm period, according to the visual resizing of the modified region, which is apparently lower than that of the original non-modified material around it.

**Figure 8:**
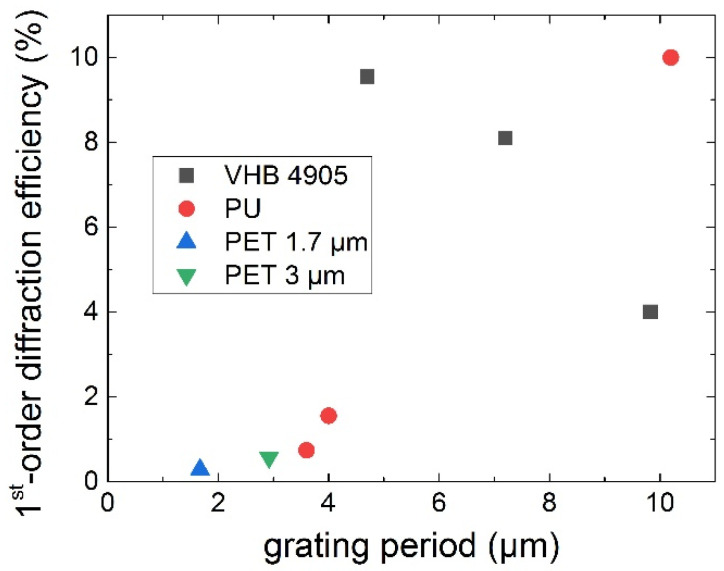
Experimentally measured 1st-order optical diffraction efficiency of the bulk shrinkable PU, PET and VHB 905 diffraction gratings at the 633 nm wavelength versus the grating periods.

**Figure 9:**
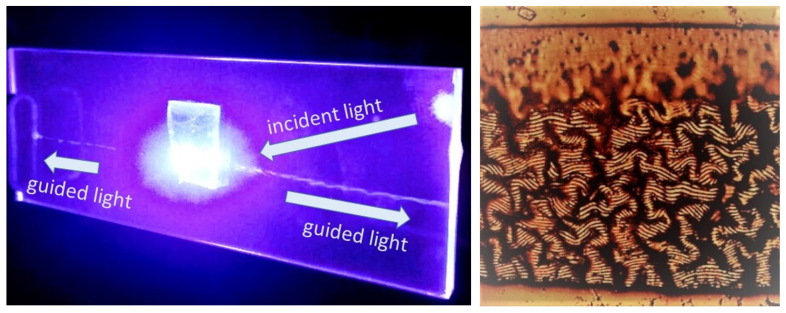
(**left**) 447 nm laser light guided in the 1.5 mm thick glass slide in the optical waveguide mode, due to total internal reflection of the blue light diffracted in the 350 nm diffraction grating made in the VHB 4905 elastomer sheet (attached to the glass in the center). (**right**) Optical image of the bulk diffraction grating in the VHB 4905 elastomer disturbed during the fs-laser exposure at the threshold energy of 28 nJ. The frame size is 400 μm × 400 μm.

Figure 8 provides the overview of the measured first-order optical diffraction efficiencies of all these considered bulk PU, PET and VHB 4905 gratings for the 633 nm laser light source as a function of their periods. Particularly, the bulk thermoplastic PU diffraction gratings demonstrated a strongly diminished diffraction efficiency—from 10% to 1%—for the smaller periods of 3–4 μm. In contrast, the bulk thermoplastic PET diffraction gratings exhibit much smaller (≈0.5–1%) but almost constant optical diffraction efficiencies (Figure 8). The most surprising fact is the rapidly raising optical diffraction efficiency trend for the elastomer VHB 4905. Since this material is highly deformable, it was 10-fold stretched prior to the fs-laser inscription of the bulk 1.7-μm grating and then shrunk till the 350 nm period. As a result, in the bulk sub-wavelength grating, we observed not the 633 nm optical diffraction patterns but, on the opposite, the waveguiding effect due to total internal reflection envisioned by the 447 nm blue laser light (Figure 9).

**Figure 10:**
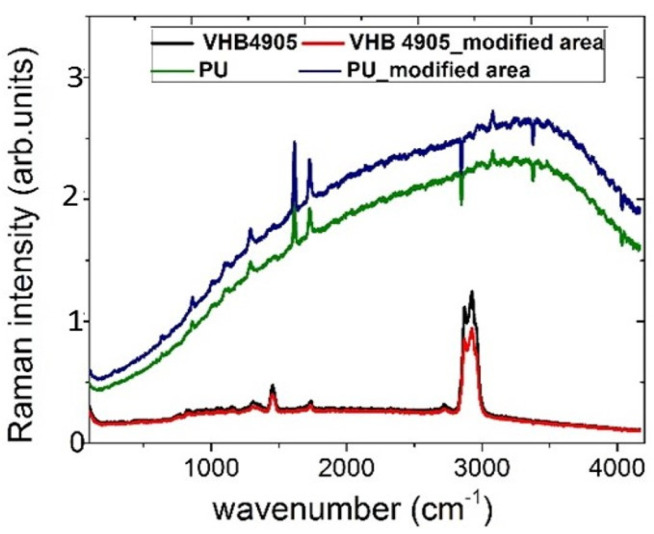
Raman spectra acquired in the non-modified and modified (inscribed diffraction grating) regions inside VHB 4905 elastomer and PU thermoplastic polymer at the 532 nm laser excitation.

Interestingly, the exposure of the elastomer VHB 4905 at the threshold fs-laser pulse energy of 28 nJ results in the irreversible multi-micron mechanical disturbance of the modified region, accompanying its 2.5-fold vertical and 1.5-fold horizontal shrinkage. Meanwhile, no considerable changes in Raman scattering intensities were observed in the non-modified and modified (inscribed) regions inside the VHB 4905 elastomer and the PU thermoplastic polymer at the 532 nm laser excitation (Figure 10), thus indicating no carbonization or other dramatic molecule-level structural modifications.

In the end, in this study, we utilized for the laser inscription in the polymers just the available 515 nm wavelength, the 0.3-ps laser pulse width and the very limited range of pulse energies, as well as the 0.25-NA and 0.65-NA focusing optics. Meanwhile, some observations reported in the literature indicate that other ultrashort-pulse laser wavelengths, shorter or longer pulse widths, spatial, temporal and spatiotemporal focusing systems, as well as other pulse-energy ranges, could be advantageous for delicate and precise micromachining inside bulk polymers in case-specific applications (for recent bibliographic sources see, for example, ref. [31].

## 4. Conclusions

In this study, we explored the still challenging fabrication of bulk diffraction optical elements in polymers, utilizing in our work in a few steps direct 515 nm fs-laser inscription in deformable elastomer VHB 905 and heat-shrinkable polymers (polyurethane and polyethylene terephthalate thermoplastics), related to mechanical relaxation of the pre-stretched elastomer or thermal shrinking of the thermoplastics along with the inscribed bulk micro-scale structures. Using as tests laser-inscribed bulk one-dimensional diffraction gratings, the scalability of their periods and diffraction efficiencies through the deformation relaxation was studied, revealing the non-linear dependence between the shrinkage and grating periods and the corresponding non-trivial dependences of their optical diffraction efficiency on the shrinkage magnitude—raising for the elastomer, constant for the polyethylene terephthalate, while diminishing for the polyurethane. The absolute optical diffraction efficiency values approach 10%, being comparable to the current holographic fabrication technologies. The bulk inscription character was demonstrated by scanning electron microscopy at 30-keV electron energy (top-view grating picture), 3D-scanning confocal photoluminescence/Raman micro-spectroscopy (photo-luminescent and Raman 3D-images) and atomic force microscopy (no surface grating relief). The obvious advantage of our method is the potentially controllable direct laser inscription of the phase profile of the diffraction element independently at each point of the magnified (micrometer) scale in the pre-stretched or pre-shrunk states. Moreover, owing to the pre-stretching and shrinkage steps, this method enables fs-laser inscription of bulk optical diffraction elements even smaller than the achieved period of 0.35 μm. Overall, this novel few-step method paves the way for facile and robust ultrashort-pulse laser inscription of bulk functional optical elements in polymeric materials for diffraction, holographic and virtual reality devices.

## Data Availability

The data supporting the reported results can be obtained from the authors upon a reasonable request.
